# Computational mass spectrometry for exposomics in non‑target screening

**DOI:** 10.1007/s00216-025-06093-7

**Published:** 2025-09-05

**Authors:** Torsten C. Schmidt, Gerrit Renner, Saer Samanipour

**Affiliations:** 1 University of Duisburg-Essen, Instrumental Analytical Chemistry, Universitaetsstr. 5, 45141 Essen, Germany; 2 Centre for Water and Environmental Research (ZWU), Universitaetsstr. 2, 45141 Essen, Germany; 3 Saer Samanipour, University of Amsterdam, Faculty of Science, Van’t Hoff Institute for Molecular Sciences, 1090 GD Amsterdam, The Netherlands

Non-target screening (NTS) in exposomics revolutionizes the way we investigate the multitude of chemicals to which humans are exposed in their environment. Instead of focusing solely on known contaminants, NTS casts a wide net, utilizing high-resolution mass spectrometry to detect any chemical features in complex samples. This approach is powerful but data-intensive – a single NTS run can produce thousands of features (peaks), yielding immense complexity and volume-comprehensive datasets. Computational data processing has thus become the critical cornerstone of exposomics: without advanced algorithms and software, it would be impossible to extract meaningful insights from these rich but unwieldy data.

As the field progresses, several significant analytical challenges have come into focus. First, researchers face an information overload problem: only a small fraction of the detected features can typically be identified or annotated, with studies often annotating just a few percent of the total features. The vast remainder constitutes a "dark" chemical space of unknowns. Second, there is a lack of standardization in how data is processed and reported – different labs and software workflows can produce divergent peak lists from the same raw data with notably low agreement. This inconsistency complicates the comparison of results across studies and undermines confidence in the findings. Relatedly, ensuring confidence in compound annotation is challenging; most tentatively identified features ultimately end up at lower confidence levels (e.g., level 3 or 4 on the Schymanski scale), meaning their structures are only partially matched or guessed.

Finally, reproducibility remains a concern: many data processing and identification workflows are implemented in closed or proprietary systems, which limits transparency and hinders independent verification. Even when the same raw data are used, non-transparent implementations can significantly influence which compounds are reported, making it challenging to reproduce findings or integrate data from different sources. These hurdles, including information overload, non-standard methods, low annotation confidence, and reproducibility issues, currently limit the application of exposomic NTS. This topical collection aims to showcase new concepts and computational strategies that empower researchers to tackle these challenges. By highlighting innovative workflows, open-source software tools, standardization efforts, nomenclature harmonization, and advances in in silico libraries, this collection illustrates how computational mass spectrometry is making exposomics more effective, reliable, and insightful. The contributions here span multiple fronts – from core data processing improvements to expanded identification capabilities – all converging on making non-target screening data more manageable, comparable, and actionable.

The advances featured in this topical collection bode well for the future of computational exposomics. As machine learning and automation become further entwined with mass spectrometry, we anticipate even more powerful tools for feature extraction, spectral prediction, and pattern recognition, which will help sift through complex datasets and flag the most critical findings. Community efforts toward open data standards, shared spectral libraries, and transparent workflows will continue to improve reproducibility and enable collaborative progress. Ultimately, combining high-quality data, ingenious algorithms, and agreed-upon standards will push exposomic NTS closer to its full potential: a routine capability to monitor the unknown unknowns in our environment and inform public health and regulatory actions accordingly.

In closing, we sincerely thank all the authors for contributing their cutting-edge research to this collection and the reviewers for their constructive evaluations, which greatly improved each article. We also thank the *Analytical and Bioanalytical Chemistry* editorial team for their support in assembling this topical collection. We hope that the insights and innovations presented here serve as a valuable resource to both new and seasoned researchers in the field, inspiring further developments in computational mass spectrometry and fostering a deeper understanding of the exposome. Each step forward in data handling, identification, and standardization brings us closer to the ultimate goal of exposomics: comprehensively charting the chemical space of our environment and its impact on humanity.

